# Anasarca, Treated by Decoction of Onions, Cured

**Published:** 1854-09

**Authors:** Ramon Uranza

**Affiliations:** Navarre; El Porvenir Medico, Madrid


*Anasarca, treated by decoction of Onions, cured.* By RAMON URANZA, of Navarre.

Manuel Goñy, native of Berriozar, æt. 16, shepherd by profession; lymphatic temperament, constitution feeble, idiosyncrasy gastro-hepatic. Has enjoyed good health until a year since, when he was attacked with a pulmonary catarrh, which confined him to bed for some days. He recovered sufficiently to resume his ordinary pursuits, though frequently troubled with cough, until the beginning of December, when his condition was described as follows:

Decubitus on left side, frequent and full pulse, heat of skin, intense thirst, cephalalgia; tongue red on the edges and tip, furred in the centre; anorexia, frothy urine, frequent dry cough and sense of pain through the whole chest.

*Diagnosis.*—Chronic pulmonary catarrh, with intense exacerbation in consequence of exposure to cold.

*Treatment.*—Venesection, barley water for diet and drink. The bleeding was repeated the next day, and a pectoral ptysan prescribed. Under the use of demulcents for six days; symptoms abated and expectoration was less painful.

*December 12th*, 8th day.—Remarked that the belly and inferior extremities had increased in volume, the fever continued, and the urinary secretion was very scanty, and the thirst very intense. Diuretics and sudorifics for four days with advantage; but on the 12th day of the case, anasarca was manifest, not only in the belly and lower extremities, but also in the upper, as well as in the face and neck.

The rapid progress of the disease, destroyed not only my confidence in the resources of science, but also that of the patient and his friends; he was left in the hands of the priest to afford him the consolations of our holy religion.

On the 16th day, the young shepherd was threatened with asphyxia, exciting the terror and admiration of beholders, on account of the enormous distention of the skin, which hindered all motion.

In this critical and dangerous condition, I remembered to have read in "El Porvenir Medico," a case of ascites in Almaden, reported by D. Juan Francisco Gallego, cured by onions and milk. My patient feeling no disposition to drink milk or eat raw onions, owing to dyspnœa, I directed a decoction of two onions of ordinary size, in a gallon of water, to be drank *ad libitum.*

Though no relief was found on the second day, the decoction was continued, and in three days, (20th day of the case,) the thirst was diminished, the flow of urine had increased, and the skin of the lower extremities was somewhat wrinkled.

On the 23d day, the abnormal volume of the belly and extremities was reduced one half, so that the patient could assume any position in bed. He had some appetite and was allowed broth. Decoction of onions continued.

On 33d day, (25th of the anasarca,) he was without fever, had a fair appetite, and no serous infiltration could be discovered. The decoction was continued, and at the end of a few days, he returned to his habitual occupation.

I should remark that I have since treated a young woman, who after a severe and long continued disease, was attacked with anasarca, with decoction of onions. In a very few day, copious diuresis occurred, and convalescence was in a short time established. But I refrain from reporting the details of this case, because other remedies were employed at the same time.

*El Porvenir Medico*, Madrid, March 15th, 1854.

